# Impact of B Cell Depletion on COVID-19 in Kidney Transplant Recipients

**DOI:** 10.3390/v15071520

**Published:** 2023-07-07

**Authors:** Naohiro Aida, Taihei Ito, Kei Kurihara, Izumi Hiratsuka, Megumi Shibata, Atsushi Suzuki, Midori Hasegawa, Takashi Kenmochi

**Affiliations:** 1 Department of Transplantation and Regenerative Medicine, School of Medicine, Fujita Health University, 1-98, Dengakugakubo, Kutsukake-cho, Toyoake 470-1192, Aichi, Japan; i-taihei@fujita-hu.ac.jp (T.I.); kurihara@fujita-hu.ac.jp (K.K.); kenmochi@fujita-hu.ac.jp (T.K.); 2 Department of Endocrinology, Diabetes, and Metabolism, School of Medicine, Fujita Health University, 1-98, Dengakugakubo, Kutsukake-cho, Toyoake 470-1192, Aichi, Japan; idumi0630@yahoo.co.jp (I.H.); megumi03@fujita-hu.ac.jp (M.S.); aslapin@fujita-hu.ac.jp (A.S.); 3 Department of Nephrology, School of Medicine, Fujita Health University, 1-98, Dengakugakubo, Kutsukake-cho, Toyoake 470-1192, Aichi, Japan; mhase@fujita-hu.ac.jp

**Keywords:** B-lymphocyte subsets, COVID-19, immunosuppression therapy, kidney transplantation, rituximab

## Abstract

Kidney transplant recipients are patients at high risk for coronavirus disease 2019 (COVID-19) due to being on immunosuppressive therapy. B cell depletion therapy, including rituximab, is an important strategy for ABO-incompatible transplants. However, knowledge about the effect of B cell depletion therapy on COVID-19 is lacking. Thirty kidney transplant recipients who developed COVID-19 were included in this study. To examine the impact of B cell depletion therapy, we retrospectively investigated the relationship between the background of the patients and the clinical outcome. Of the 30 patients, 13 received B cell depletion therapy. The median time between transplant and onset of COVID-19 was 6.1 years after transplantation; however, nine cases remained markedly depleted of CD19(+) cells (<4.0%). The patients were assigned to the normal (*n* = 21) and depletion groups (*n* = 9). Progression rates in the depletion and normal groups were 55.6% and 9.5%, respectively (*p* = 0.014). Furthermore, the survival rate was significantly lower in the depletion group (100% in the normal group vs. 66.7% in the depletion group; *p* = 0.021). B cell depletion therapy may have long-term effects and increase the risk of COVID-19 in kidney transplant recipients.

## 1. Introduction

Since 2019, the severe acute respiratory syndrome coronavirus-2 has been widely spreading. The current strategy involves immunity acquisition by vaccination and appropriate therapy after disease onset [1]. Clinical management is important, especially in high-risk patients, as severe illness from coronavirus disease 2019 (COVID-19) can lead to death.

Post-transplant patients are considered high-risk because they receive immunosuppressive therapy. Although the short-term efficacy of vaccination has been demonstrated in the population [2,3], other reports indicate that 46% of post-transplant recipients do not develop antibodies after two doses of mRNA vaccine [4]. Booster vaccinations are being attempted to increase the antibody acquisition rate; however, individualized management according to the patient’s status after onset is also considered necessary. Radcliffe et al. reported that antiviral therapy or neutralizing monoclonal antibody therapy is useful for reducing mortality and hospitalization rates in kidney transplant patients with mild COVID-19 who can be treated on an outpatient basis [5]. This also indicates that some patients improve without active treatment and that there are differences in the risks among kidney transplant recipients. Age, hypertension, and diabetes are additional risk factors; however, one possibility is that the degree of risk differs depending on the immunosuppressive therapy content. Montero et al. reported that calcineurin inhibitors (CNIs) negatively impact the clinical course of COVID-19 [6]. However, the evidence for other drugs is insufficient.

B cell depletion therapy is an important therapy for ABO-incompatible kidney transplantation. In Japan, due to a shortage of donors, the proportion of ABO-incompatible transplantations is 20% of that of living donor kidney transplantation [7]. These recipients need to receive strong immunosuppressive therapy combined with B cell depletion therapy, including rituximab (Rmab), and are, thus, expected to be at an even higher risk as compared to ABO-compatible transplantation recipients. Therefore, it is necessary to examine COVID-19 infection in more detail in kidney transplant patients undergoing B cell depletion therapy.

This study was designed to retrospectively investigate the clinical course of COVID-19 in kidney transplant patients and analyze the impact of B cell depletion therapy.

## 2. Materials and Methods

### 2.1. Patients

A total of 28 adult kidney transplant recipients (22 patients who underwent kidney transplantation and 6 patients who underwent simultaneous pancreas and kidney transplantation) with symptomatic COVID-19 were included in this study.

The participants were selected according to the following conditions: (1) Kidney transplant recipients undergoing follow-up at our department. (2) Those who developed COVID-19 between February 2022 and December 2022 and completed treatment. This period was determined according to the prevalence of the Omicron strain in Japan. In addition, patients whose B cell subset was not measured before disease onset were excluded. This exclusion criterion excluded two patients who did not receive B cell depletion therapy.

### 2.2. Study Items

Patient background data such as age, sex, body mass index (BMI), transplantation procedure, immunosuppressive therapy, and vaccination were collected. Furthermore, clinical data such as blood examination and computed tomography images taken during hospital visits were also collected, and outcomes were analyzed. 

### 2.3. Medical Practice for COVID-19

Patients were able to receive either the BNT162b2 or mRNA-1273 vaccine. In this study, the number of vaccinations was examined regardless of the type of vaccine administered. All patients in this study had symptomatic COVID-19 with a confirmed diagnosis by polymerase chain reaction or antigen testing. Patient severity was determined based on the World Health Organization criteria [8]. They were classified according to their presentation state at the time of the visit (oxygen saturation, presence of pneumonia, and other clinical data). Patients with oxygen saturation ≥ 96% or more without pneumonia were considered to have mild disease, those with pneumonia but no decrease in oxygen saturation were considered to have moderate disease, and those with pneumonia and decreased oxygen saturation were considered to have severe disease. None of the patients had severe respiratory failure or septic shock, which are considered critical diseases.

### 2.4. Treatment for COVID-19

All symptomatic COVID-19 transplant patients were treated in the hospital; outpatient treatment was not allowed. Remdesivir was administered even in cases of mild disease, and patients with moderate to severe disease received dexamethasone and tocilizumab. Available monoclonal antibody preparations were rarely used due to their poor efficacy against Omicron strains. Immunosuppressants were reduced according to severity. Specifically, MMF was reduced even for patients with mild disease. Patients with moderate or severe disease discontinued MMF. Discontinuing CNI or everolimus were also considered for patients with severe disease. Steroids were basically continued.

### 2.5. Immunological Status at Transplantation and Immunosuppressive Protocol

Of the 22 kidney transplant recipients, eight underwent ABO-incompatible living donor kidney transplantation. For ABO-incompatible transplantation, 200 mg/body weight of Rmab was administered intravenously 2 weeks before transplantation, and oral mycophenolate mofetil (MMF) was started. Of the remaining 16 patients, three patients received the same dose of Rmab as the ABO-incompatible transplantation for various reasons. The post-operative immunosuppressive protocol was the same for all patients and included tacrolimus, MMF, and steroids. All patients received basiliximab as an induction therapy. The trough tacrolimus level was controlled at 5–8 ng/mL for the first 3 months and 3–8 ng/mL thereafter. In some patients, MMF was reduced, and everolimus was added because of the frequency of infections or side effects. A single dose of 200 mg/body weight was administered if Rmab was used for post-operative antibody-mediated rejection.

### 2.6. B Cell Population

All patients had lymphocyte subsets measured using flow cytometry in 2021 to examine the impact of immunosuppressive therapy as part of a post-transplant follow-up. To investigate the effect of B cell depletion therapy, the ratio of CD19(+) cells was examined. B cell depletion was defined as the condition when the CD19(+) cell rate was 4.0% or less. Furthermore, to examine antibody production ability, serum IgM and IgG antibody titers were measured using turbidimetric immunoassay on the same day.

### 2.7. Ethical Considerations

This study was conducted in accordance with the Declaration of Helsinki and approved by the Ethics Committee of Fujita Health University (HM22-436). The contents of this study were posted on the web page of Fujita Health University and in the outpatient clinic, and all participants were provided with the opportunity to withdraw from the study.

### 2.8. Statistical Analysis

Continuous variables were expressed as medians (minimum–maximum). The Mann–Whitney U test was used to compare the groups. Fisher’s exact test was used to compare the frequencies. All statistical analyses were performed using EZR software (Saitama Medical Center, Jichi Medical University, Saitama, Japan) [9]. Statistical significance was set at *p* < 0.05.

## 3. Results

### 3.1. Comparison by Disease Severity

Twenty-eight patients developed COVID-19 after February 2022, when the Omicron strain was prevalent. There were two deaths in the period. Eighteen patients (64.3%) had mild disease at the hospital visit; 10 patients had pneumonia, of which four patients (14.3%) had severe disease requiring oxygen. 

Table 1 shows the backgrounds of patients of each severity at the hospital visit. The median age was 54.5 (34–74) years for patients with mild disease, 48 (36–71) years for those with moderate disease, and 75.5 (49–77) years for those with severe disease (*p* = 0.075). There was no significant difference in the proportion of operative procedures. The time between transplantation and COVID-19 onset was 5.1 (1.6–14.9) years for patients with mild disease, 3.2 (1.4–7.1) years for those with moderate disease, and 3.9 (1.2–9.2) years for those with severe disease, and there was no difference in the severity. Among the immunosuppressants, CNI, MMF, and everolimus were not associated with severity. However, three of the four patients (75.0%) with severe disease had a history of B cell depletion therapy, which was higher than that of mild and moderate disease. Regarding blood examinations, lactate dehydrogenase (LDH) levels and C-reactive protein (CRP) levels were significantly higher with higher disease severity. The proportion of patients with B cell depletion tended to be higher with increasing severity (mild vs. moderate vs. severe disease: 4/18 (22.2%) vs. 2/6 (33.3%) vs. 2/4 (50.0%), *p* = 0.52). Furthermore, the IgG titer tended to lower with increasing disease severity (mild vs. moderate vs. severe disease: 1129 (440–1830) mg/dL vs. 903.5 (757–1098) mg/dL vs. 752 (656–869) mg/dL, *p* = 0.12). There was no difference in kidney graft function at onset (mild vs. moderate vs. severe disease: 1.3 (0.6–3.4) mg/dL vs. 0.9 (0.6–1.8) mg/dL vs. 1.2 (1.0–2.5) mg/dL, *p* = 0.39). Disease progression after treatment was seen in 1/18 (5.6%) cases of mild disease, 1/6 (16.7%) cases of moderate disease, and 3/4 (75%) cases of severe disease (*p* < 0.01). None of the patients with mild disease and moderate disease required an intensive care unit (ICU) stay. In contrast, 4/4 (100%) cases with severe disease required an ICU stay (*p* < 0.01). The duration of hospitalization also tended to be longer with higher disease severity (mild vs. moderate vs. severe disease: 6 (3–20) days vs. 15 (8–38) days vs. 35.5 (16–38) days, *p* < 0.01). The patient survival was 100% (18/18) for mild disease, 100% (6/6) for moderate disease, and 50% (2/4) for severe disease (*p* < 0.01). Both of the two patients who died were severe disease, over 65 years of age, and had a history of rituximab use. In contrast, the remaining two patients with severe disease were younger age or had no history of rituximab use.

### 3.2. Association between B Cell Depletion Therapy and COVID-19

Our findings so far suggest that B cell depletion therapy affects the clinical course of COVID-19 in post-transplant patients. Therefore, these 12 cases were further examined. Table 2 shows the data from 12 patients treated with B cell depletion therapy sorted by the percentage of CD19(+) cells.

The 12 patients who underwent B cell depletion therapy did so due to ABO-incompatible transplantation in eight cases, antiphospholipid antibody syndrome in one case, cryoglobulinemia in one case, and intensification of induction therapy in one case. One patient underwent Rmab treatment for antibody-mediated rejection. The median time from B cell depletion therapy to COVID-19 onset was 6.2 (1.6–14.9) years. All the patients received three or more immunosuppressants (CNIs and steroids plus MMF and/or everolimus). Of the 12 patients who received B cell depletion therapy, eight had CD19(+) cells less than 4% and were considered to have B cell depletion. The median years after Tx for these eight patients were 4.5 (1.6–14.9) years. The remaining four patients had a normal ratio of CD19(+) cells. Five patients had moderate or severe disease, four of whom had CD19(+) cell depletion. Disease progression was observed in five cases, four of whom had CD19(+) cell depletion. Moreover, two patients who died showed markedly CD19(+) cell depletion.

**Figure 1:**
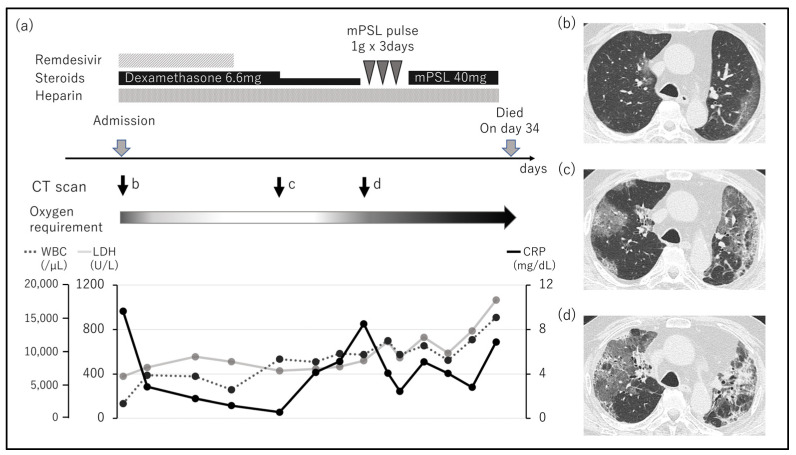
The clinical course of a patient who died of COVID-19. (**a**) The clinical course of a patient who died of COVID-19 caused by the Omicron variant. On arrival, the patient was diagnosed with severe disease. Five days of remdesivir and 7 days of high-dose dexamethasone improved respiratory disorder and eliminated oxygen requirement. COVID-19 exacerbated after dose reduction in dexamethasone. Despite intensive therapy including steroid pulse, the patient died on day 34. (**b**) CT images on arrival. A focal ground-glass opacity is observed in the upper left lobe. (**c**) CT images on day 10. The ground-glass opacity had spread. It was diagnosed to be a healing process. (**d**) CT images on day 21. Widespread ground-glass opacities were diagnosed as the progression of COVID-19.

Figure 1a shows the clinical course of a patient who died of COVID-19 caused by the Omicron variant. The patient was a 77-year-old male with 0.6% CD19(+) cells. He was diagnosed with severe disease on arrival because the patient had pneumonia and required oxygen (Figure 1b). Tacrolimus and MMF were immediately discontinued, and treatment with remdesivir, dexamethasone, and heparin was initiated. Five days of remdesivir and 7 days of high-dose dexamethasone improved respiratory disorder and eliminated oxygen requirement. After dexamethasone was reduced to half dose, CT images on day 25 showed an exacerbation of ground-glass opacities (Figure 1d). Despite intensive therapy including steroid pulse, the patient died on day 34. Thus, it was difficult to control disease progression in some patients with B cell depletion, even with appropriate therapy.

### 3.3. Association between B Cell Depletion and Clinical Course

These studies suggest a relationship between B cell depletion and the clinical course of COVID-19. Therefore, the 28 patients were again divided into two groups based on the percentage of CD19(+) cells, and a comparative study was conducted. Patients with a CD19(+) cell count ≥ 4.0% were classified into the normal group and those with a CD19(+) cell count < 4.0% into the depletion group.

Table 3 shows the background of the patients with B cell depletion. Patients with B cell depletion were significantly older than those in the normal group (normal group vs. depletion group: 52 (34–75) years vs. 70 (41–77) years, *p* = 0.039). They had a history of either Rmab use or splenectomy, which resulted in a high rate of ABO-incompatible kidney transplantation in the Tx procedure. There were no differences between groups in the use of immunosuppressants other than Rmab. Almost all patients received vaccination; there was no difference in vaccination frequency between the two groups (Normal group vs. Depletion group: 19/20 cases vs. 8/8 cases). Comparing blood examination, WBC counts and CRP level at the time of visit were comparable between the Depletion group and the Normal group. LDH level was significantly higher in the Depletion group (Normal group vs. Depletion group: 240 (141–623) U/L vs. 306.5 (236–403) U/L, *p* = 0.038). Furthermore, peak CRP levels during hospitalization were significantly higher in the Depletion group (Normal group vs. Depletion group: 2.1 (0.1–17.8) mg/dL vs. 7.3 (4.6–12.1) mg/dL, *p* = 0.032). IgG titer was lower in the depletion group, but there was no significant difference. Although there was no significant difference in the severity at the visit, the proportion of patients with moderate to severe disease was higher among those with B cell depletion. Furthermore, disease progression was observed in four of eight patients with B cell depletion, which was significantly higher than the number in the normal group (*p* = 0.015). The length of hospital stay was significantly longer in patients with B cell depletion (normal group vs. depletion group: 7 (3–38) vs. 16 (7–35) years, *p* = 0.034), and the survival rate of patients with B cell depletion was lower than that in the normal group (normal group vs. depletion group: 100 (20/20) vs. 75.0 (6/8) %, *p* = 0.074).

## 4. Discussion

This was a retrospective study on COVID-19 in transplant recipients. This study revealed that B cell depletion therapy, including Rmab treatment, has a negative impact on the clinical course of COVID-19. Most importantly, B cell suppression continues long after transplantation. In general, the effects of Rmab have been shown to last 3 months to a year in other diseases and longer in kidney transplantation [10]. This study suggests that the effects may last over 5 years to suppress B cells. Sustained effects beyond expectations may endanger post-transplant patients. This is not only the effect of Rmab alone but also the effect of maintenance immunosuppressive therapies, such as MMF and everolimus, following Rmab administration. These two agents are known to suppress B cells and strongly reduce antibody production [11], which may prolong the effects of B cell depletion therapy. Although effective in suppressing rejection, they can be very threatening during such a pandemic. It is necessary to recognize that kidney transplant patients who undergo depletion therapy may be at particular risk.

There are two concerns regarding B cell suppression during the COVID-19 pandemic. First, it may have reduced the effects of vaccination. As is well known, B cells are closely related to the acquisition of antibodies by vaccination [12]. It has been reported that post-transplant patients exhibit an extremely low antibody acquisition rate compared with healthy individuals and patients with other chronic kidney diseases [13]. Immunosuppressive therapy such as MMF has been reported to be a related factor [14,15]. Studies on Rmab are limited because of its low usage rate; however, Haskin et al. reported that the antibody acquisition rate was low in kidney transplant patients treated with Rmab [16]. A meta-analysis by Manothummetha et al. also indicated that Rmab use within 1 year was associated with a risk of decreased antibody acquisition rate [17]. Takai et al. showed that the antibody acquisition rate is low within 2 years after Rmab administration in kidney transplant patients [18]; however, the longer-term effects of Rmab have not been investigated. It should be noted that B cells may be suppressed for nearly 5 years with adequate maintenance therapy, as revealed in this study.

The second factor is its effect on the immune response after infection. There have been reports of aggravation in Rmab-administered patients, including other diseases [19,20,21]. Similar to T cells, B cells play an important role in the adaptive immune responses after viral infection. The same was true for COVID-19 [22]. In addition, T cell and low lymphocyte counts have been identified as important risk factors for severe disease, suggesting that B cells are as important as T cells in the COVID-19 infection phase. Excessive immune responses are involved in COVID-19 exacerbation [23], and early viral control is critical for avoiding this. B cell suppression may indicate a reduction in humoral immunity and localization of its effects in this critical aspect.

B cell suppression has a negative impact on the clinical course of COVID-19, however, refusal to use Rmab, which deprives the patient of the opportunity for ABO-incompatible transplantation, increases the risk of life-threatening renal failure. The best solution is to establish methods to prevent such patients from developing severe diseases. In Japan during the COVID-19 pandemic, vaccination was recommended for transplant recipients as in other countries. However, renal transplant recipients, especially those who have received B cell depletion therapy, may have a low rate of antibody acquisition from the vaccine. This is a disadvantage in a pandemic situation where rapid antibody acquisition is desired. One solution would be the prophylactic administration of antibody preparations. Neutralizing antibodies have been used as therapeutic agents since the early days of the COVID-19 pandemic to prevent the disease from becoming severe. Prophylactic administration of tixagevimab and cilgavimab has proven efficient against the Omicron BA1 and BA2 [24,25]. Furthermore, in the PROVENT study, a booster dose was given half a year later, and efficacy was shown for 1 year [24]. Thus, regular antibody administration may be effective in high-risk patients. Since the effects of B cell depletion therapy are longer than expected owing to maintenance immunosuppressive therapy, assessing the proportion of B cells may be a criterion for drug administration. In addition, it is noted that the effect of neutralizing antibodies was limited to certain strains [26]. Casirivimab and imdevimab were no longer effective against the Omicron strain. In the future, administering different antibody preparations for strain mutations may be necessary.

This study has some limitations. First, the number of cases was limited, and the statistical explanation may be weak. Therefore, great care should be taken in interpreting the results, making it difficult to study B cell suppression and age. In this study, the B cell suppression group tended to be elderly. As has been previously reported, advanced age is one of the risk factors for COVID-19, and it may have resulted in severe outcomes. However, B cell suppression should be recognized as a risk, as older patients not undergoing this therapy had normal B cell ratios and did not die. Second, this is a retrospective study. The results of this study were only for those who developed the symptomatic disease, and there is a possibility that the target patients are biased. Furthermore, this study did not assess T cell activity, which influences the clinical course of COVID-19. This is because the immunocompetence data used in this study were limited to standard post-transplant examinations. Therefore, further research is needed to obtain more scientific clarification. This research also has strengths. Although this study showed that Rmab administration might suppress B cells in the long term, this fact can be recognized as important even if the number of cases is small. There is no other research on the long-term effect of B cell depletion therapy.

## 5. Conclusions

B cell depletion therapy and maintenance of immunosuppressive therapy may result in long-term B cell suppression in kidney transplant patients. Patients with B cell depletion therapy should require appropriate therapeutic intervention, such as administration of neutralizing antibodies, because B cell suppression adversely affects the clinical course of COVID-19.

## Figures and Tables

**Table 1:** Background of all patients.

	All (*n* = 28)	Mild (*n* = 18)	Moderate (*n* = 6)	Severe (*n* = 4)	*p*- Value
Age (year)	54.5 (34–77)	54.5 (34–74)	48 (36–71)	75.5 (49–77)	0.075
Sex (male, %)	16 (57.1)	11 (61.1)	2 (33.3)	3 (75.0)	0.36
BMI (kg/m^2^)	22.2 (15.4–32.5)	22.2 (15.4–29.9)	22.1 (19.9–31.6)	22.6 (20.1–32.5)	0.90
Tx Procedure (%)					0.72
ABO-compatible KTx	14 (50.0)	8 (47.4)	4 (66.7)	2 (50.0)	
ABO-incompatible KTx	8 (28.6)	5 (15.2)	1 (16.7)	2 (50.0)	
SPK	6 (21.4)	5 (15.2)	1 (16.7)	0 (0.0)	
Post-transplant Years	4.3 (1.2–14.9)	5.1 (1.6–14.9)	3.2 (1.4–7.1)	3.9 (1.2–9.2)	0.56
Days after onset	1 (0–12)	1 (0–4)	1.5 (0–12)	1.5 (0–6)	0.59
Immunosuppressants					
CNI					1.0
Tacrolimus	26 (92.9)	16 (88.9)	6 (100)	4 (100)	
Cyclosporine	2 (7.1)	2 (11.1)	0 (0.0)	0 (0.0)	
MMF	23 (80.0)	16 (88.9)	4 (66.7)	3 (75.0)	0.37
Everolimus	8 (28.6)	5 (27.8)	2 (33.3)	1 (25.0)	0.95
Steroid	28 (100)	19 (100)	8 (100)	4 (100)	1.0
B cell depletion therapy	12 (42.9)	7 (38.9)	2 (33.3)	3 (75.0)	0.50
Blood Examination					
WBC (×10^3^/µL)	5.8 (3.0–12.2)	5.8 (3.0–12.1)	5.7 (4.1–8.3)	8.2 (5.6–12.2)	0.16
LDH (U/L)	268 (141–623)	237 (141–623)	285 (237–397)	320 (303–403)	0.059
CRP (mg/dL)	1.2 (0.1–17.0)	1.0 (0.1–2.9)	2.5 (0.5–7.4)	10.9 (7.2–17.0)	<0.01
Peak CRP (mg/dL)	3.2 (0.1–25.6)	1.5 (0.1–7.1)	5.0 (0.7–25.6)	17.0 (9.7–23.2)	<0.01
D-dimer (µg/mL)	0.9 (0.5–3.9)	0.8 (0.6–3.7)	1.5 (1.1–3.9)	1.7 (0.5–3.6)	0.13
Cre (mg/dL)	1.2 (0.6–3.4)	1.3 (0.6–3.4)	0.9 (0.6–1.8)	1.2 (1.0–2.5)	0.39
B cell depletion (%)	8 (28.6)	4 (22.2)	2 (33.3)	2 (50.0)	0.52
IgG (mg/dL)	926.5 (440–1830)	1129 (440–1830)	903.5 (757–1098)	752 (656–869)	0.12
IgM (mg/dL)	64.5 (12–198)	62 (27–198)	66 (38–156)	53 (12–125)	0.67
Outcome					
Progression (%)	5 (17.9)	1 (5.6)	1 (16.7)	3 (75.0)	<0.01
ICU stay (%)	4 (14.3)	0 (0)	0 (0)	4 (100)	<0.01
Hospitalization days	9 (3–38)	6 (3–20)	15 (8–27)	35.5 (16–38)	<0.01
Survival (%)	26 (92.9)	18 (100)	6 (100)	2 (50.0)	<0.01

BMI; body mass index, Tx; transplantation, KTx; kidney transplantation, CNI; calcineurin inhibitors, MMF; Mycophenolate mofetil, Rmab; rituximab, ICU; intensive care unit.

**Table 2:** Characteristics of patients with B cell depletion therapy ordered by CD19(+) cell counts.

Age/ Sex	Immunosuppress Protocol	Years After Tx	Cre (mg/dL)	CD19(+) Cells (%)	Severity at Visit	Hospitalization Days	Progression	Survival
76/M	R + T, M, S	6.3	2.5	0.3	Severe	16	Yes	Dead
69/M	R + T, M, E, S	1.6	1.3	0.3	Mild	7	No	Alive
77/M	R + T, M, S	1.6	1.0	0.6	Severe	35	Yes	Dead
44/F	R + T, E, S	6.1	1.3	0.9	Moderate	27	Yes	Alive
71/M	R + T, M, S	7.1	0.8	0.9	Moderate	16	No	Alive
41/M	R + T, E, S	2.9	3.4	1.0	Mild	20	No	Alive
65/F	SX + T, M, S	14.9	1.6	1.4	Mild	12	Yes	Alive
74/F	R + C, M, E, S	2.1	0.7	1.6	Mild	8	No	Alive
64/F	R + C, M, E, S	6.2	1.5	4.3	Mild	5	No	Alive
52/M	R + T, M, S	6.9	1.2	8.2	Mild	6	No	Alive
45/M	R + T, M, S	8.3	1.5	11.0	Mild	8	No	Alive
49/F	R + T, M, S	9.1	1.1	16.8	Severe	38	Yes	Alive

R; rituximab, SX; splenectomy, everolimus, M; mycophenolate mofetil, S; steroid.

**Table 3:** Characteristics of patients with B cell depletion.

Factor	Normal (*n* = 20)	Depletion (*n* = 8)	*p*-Value
Age (years)	52 (34–75)	70 (41–77)	0.039
Sex (male, %)	11 (55.0)	5 (62.5)	1.0
BMI	22.4 (17.1–32.5)	21.2 (15.4–31.6)	0.48
Tx procedure (%)			0.032
ABO-compatible KTx	11 (55.0)	3 (37.5)	
ABO-incompatible KTx	3 (15.0)	5 (62.5)	
SPK	6 (30.0)	0 (0)	
Tx to onset (years)	4.3 (1.2–9.2)	4.5 (1.6–14.9)	1.0
Vaccination (%)	19 (95.0)	9 (100)	
Immunosuppressant (%)			
CNI			0.50
Tacrolimus	19 (95.0)	7 (87.5)	
Cyclosporine	1 (5.0)	1 (12.5)	
MMF	17 (85.0)	6 (75.0)	0.61
Everolimus	4 (20.0)	4 (50.0)	0.17
Rituximab	4 (20.0)	7 (87.5)	<0.01
Splenectomy	0 (0)	1 (12.5)	
Blood Examination			
WBC (×10^3^/µL)	5.6 (3.0–12.2)	6.9 (4.6–12.1)	0.15
LDH (U/L)	240 (141–623)	306.5 (236–403)	0.038
CRP (mg/dL)	1.2 (0.1–17.0)	2.1 (0.4–7.6)	0.28
PeakCRP (mg/dL)	2.1 (0.1–17.8)	7.3 (4.6–12.1)	0.032
D-dimer (µg/mL)	0.8 (0.5–3.9)	1.5 (0.7–3.7)	0.16
Cre (mg/dL)	1.2 (0.6–2.6)	1.3 (0.7–3.4)	0.49
IgG (mg/dL)	1032 (653–1830)	888.5 (440–1098)	0.33
IgM (mg/dL)	64.5 (27–198)	61 (12–126)	0.56
Outcome			
Severity at visit (%)			0.50
Mild	14 (70.0)	4 (50.0)	
Moderate	4 (20.0)	2 (25.0)	
Severe	2 (10.0)	2 (25.0)	
Progression (%)	1 (5.0)	4 (50.0)	0.015
ICU stay (%)	2 (10.0)	2 (25.0)	0.56
Hospitalization days(days)	7 (3–38)	16 (7–35)	0.034
Survival (%)	20 (100)	6 (75.0)	0.074

SPK; simultaneous pancreas and kidney transplantation, CNI; calcineurin inhibitors, MMF; Mycophenolate mofetil.

## Data Availability

The data presented in this study are available on request from the corresponding author. The data are not publicly available due to restrictions because of their containing information that could compromise the privacy of research participants.
